# Effect of Spinetoram Stress on Midgut Detoxification Enzyme and Gene Expression of *Apis cerana cerana* Fabricius

**DOI:** 10.3390/insects16050492

**Published:** 2025-05-04

**Authors:** Lin Chen, Tianjun He, Linglong Ding, Xinyan Lan, Jiahao Sun, Xiaoheng Xu, Huafen Wu, Dayun Zhou, Zhichu Huang, Tianxing Zhou, Xiaoling Su, Limin Chen

**Affiliations:** 1 Lishui Institute of Agriculture and Forestry Sciences, Lishui 323000, China; fay321@126.com (L.C.); baiyun12_12@163.com (T.H.); 2022205014@stu.njau.edu.cn (J.S.); r05626026@163.com (X.X.); whf761002@163.com (H.W.); lssnlkyzdy@163.com (D.Z.); 13757809816@163.com (T.Z.); 2 College of Ecology, Lishui University, Lishui 323000, China; dllong0607@163.com (L.D.); lanxya4086@163.com (X.L.); 3 Jinhua Academy of Agricultural Sciences, Jinhua 321000, China; zhichu@zju.edu.cn

**Keywords:** SPI toxicity, honey bee, midgut damage, enzyme inhibition

## Abstract

This study aimed to evaluate the impact of Spinetoram (SPI) on the midgut of *Apis cerana cerana* Fabricius pupae, emphasizing detoxifying enzyme activity, gene expression, and morphological alterations. Pupae were subjected to SPI at LC_20_ and LC_50_ concentrations, and the midgut was evaluated using morphological assessment, detoxifying enzyme assays, and transcriptome analysis utilizing gene ontology and Kyoto Encyclopedia of Genes and Genomes (KEGG) enrichment. Transcriptome analysis revealed 32 differentially expressed genes (DEGs) that were common to both the LC_20_ vs. control (CK) and LC_50_ vs. CK comparisons, along with 24 DEGs unique to the LC_20_ vs. CK comparison and 76 DEGs unique to the LC_50_ vs. CK comparison. KEGG pathway analysis indicated the substantial enrichment of pathways associated with drug metabolism, xenobiotic metabolism, and amino acid metabolism, implying disruptions in detoxification mechanisms and broader metabolic imbalances resulting from SPI exposure. Morphological analysis showed a normal midgut structure in the control group, while significant damage was observed in the LC_20_ group, and severe degeneration was observed in the LC_50_ group. Detoxification enzyme assays revealed that the activities of cytochrome P450, glutathione S-transferase, and carboxylesterase significantly increased at LC_20_ (*p* < 0.05), indicating an initial induction of detoxification responses; however, they declined at LC_50_, suggesting enzyme inhibition or midgut damage. The activity of acetylcholinesterase markedly diminished at both LC_20_ and LC_50_ (*p* < 0.05), with a more substantial decline observed at LC_50_, suggesting possible neurotoxicity. These findings indicate that SPI exposure causes substantial alterations in midgut morphology, detoxifying enzyme activity, and gene expression in *Apis cerana cerana* Fabricius pupae, underscoring the insecticide’s detrimental impact on honey bee health.

## Simple Summary

The current study investigated how a common pesticide called Spinetoram (SPI) affects the Asian honey bee. We exposed pupae to different SPI doses and discovered serious harm at both low and high concentrations. The midguts of the bees and the detoxifying organ showed severe damage, starting with swelling at low doses and progressing to complete tissue breakdown at higher doses. We found that the bees’ bodies initially tried to fight the pesticide by boosting detox enzymes like cytochrome P450, but this system became overwhelmed at higher SPI levels, causing enzyme activity to crash. Alarmingly, SPI also dramatically reduced the activity of a key brain enzyme (acetylcholinesterase), indicating neurotoxic effects that could impair bee behavior and survival. Genetic analysis revealed SPI disrupted genes involved in metabolism, detoxification, and immune responses, potentially making bees more vulnerable to other environmental stresses. Our findings suggest that even sublethal SPI exposure stresses honey bee pupae, while higher doses cause irreversible damage to their digestive and nervous systems. Due to their crucial role as pollinators, these results raise significant concerns about using SPI in agriculture near bee habitats. This study highlights the need for careful pesticide management to protect these ecologically vital insects and maintain healthy ecosystems.

## 1. Introduction

Honey bees (*Apis* spp.) serve as essential pollinators for numerous crops and play a critical role in maintaining biological diversity. In recent decades, global declines in honey bee populations have been reported across Asia, the United States, and Europe [1,2,3]. This decline is primarily attributed to a combination of factors, including pathogens, pesticides, climate change, habitat loss, and poor management practices, all of which may act individually or synergistically [4]. A major contributor to colony losses is pesticide exposure [2,3,4,5,6,7,8]. Honey bee larvae are particularly vulnerable, with numerous studies demonstrating that pesticide exposure increases larval mortality, reduces hatching rates, and induces structural abnormalities in organs such as the midgut, Malpighian tubules, and mushroom bodies. It can also lead to deformities in wings and antennae [8,9,10]. Furthermore, pesticide exposure can result in cellular, physiological, and morphological damage by modulating gene expression and interfering with enzymatic activity [3,9,11,12].

Understanding how different insecticides affect honey bees is crucial for effective risk assessment. Spinetoram (SPI), a semi-synthetic derivative of the spinosyn class (Group 5, IRAC classification), represents a novel class of insecticides with a broad-spectrum mode of action [13,14,15]. Spinosyns, including Spinosad, are glycosylated macrolactones that are produced from the fermentation of the actinobacterial species *Saccharopolyspora spinosa*. Spinosad disrupts insect nervous systems, primarily by targeting γ-aminobutyric acid (GABA)-gated ion channels and nicotinic acetylcholine receptors (nAChRs). It specifically binds to the nAChRα6 subunit, triggering disruptions in calcium signaling, lysosomal function, and the generation of reactive oxygen species (ROS), ultimately causing oxidative stress and mitochondrial dysfunction. These combined effects lead to neurodegeneration and cellular damage in exposed insects.

SPI is a systemic insecticide that is widely applied to protect crops against pests such as lepidopterans, coleopterans, and thysanopterans, owing to its potent efficacy [3,16,17,18,19,20]. According to the Pesticide Properties DataBase, SPI has an acute contact LD_50_ of 0.024 µg/bee and an acute oral LD_50_ of 0.14 µg/bee in honey bees [21]. As per the US Environmental Protection Agency (EPA), SPI is toxic to bees, especially when applied during blooming periods or when residues persist on flowering plants or weeds [22]. Acute toxicity tests have shown that both contact and oral exposure to SPI significantly increase bee mortality in a dose-dependent manner. The LD_50_ values of 0.024 µg/bee (contact) and 0.14 µg/bee (oral) further highlight its hazard potential. Consequently, it is recommended to avoid using SPI in areas where bees are actively foraging or where drift to blooming plants is likely [8,23].

Given the acute toxicity of SPI and its potential to persist in the environment, it is critical to assess its sublethal impacts on *Apis cerana cerana*, a native Asian honey bee species [24,25,26]. While prior studies have focused on SPI-induced mortality [8], more detailed investigations are needed to understand its specific effects on the midgut of *Apis cerana cerana* (Fabricius) [10]. The midgut is a vital organ that is responsible for digestion, detoxification, and xenobiotic metabolism, making it a prime target for evaluating pesticide toxicity. This study investigates the effects of SPI on midgut morphology, detoxifying enzyme activity (CYP450, GST, CarE, and AChE), and gene expression in *Apis cerana cerana* (Fabricius). Biochemical markers, now widely used in toxicity evaluations, were employed to assess the physiological responses of honey bees to SPI exposure and to elucidate detoxification mechanisms [27,28,29,30].

Moreover, transcriptome analysis using next-generation sequencing was conducted to explore global gene expression changes in the midgut of *Apis cerana cerana* following SPI treatment. This molecular approach enables the identification of pesticide-induced disruptions in energy metabolism and detoxification pathways. Using whole-transcriptome sequencing, we compared gene expression profiles between control bees and those exposed to SPI at LC_20_ and LC_50_ concentrations (LC = lethal concentration), identifying differentially expressed genes (DEGs). Subsequent gene ontology (GO) and Kyoto Encyclopedia of Genes and Genomes (KEGG) pathway enrichment analyses were performed to gain insights into the molecular mechanisms underlying SPI-induced stress responses, thereby contributing to a deeper understanding of the honey bee’s molecular defense against xenobiotic stressors.

## 2. Materials and Methods

### 2.1. Insect Samples

The colonies of the Chinese honey bee (*Apis cerana cerana* Fabricius) utilized in the experiment were raised at the Lishui Institute of Agriculture and Forestry Sciences from June to September 2024. The larvae were raised in specialized perforated 24-well plastic rearing boxes (15 × 15 × 15 cm) at the apiary according to Peng et al. [31] and maintained in an incubator with regulated temperature and humidity for 24 h. The humidity was maintained at 75% until pupal stage development. The honey bees at the pupal stage were brought from the apiary into the laboratory for further experiments. The pupal brood cells capped by other bees (a capped spleen) from each of the four healthy colonies were maintained in an incubator at 35 °C and 75% relative humidity (MGC-1500BP-2, Shanghai Yiheng Scientific Instrument Co., Ltd., Shanghai, China) for 12 h. After 12 h, 500 pupae from the three hive spleens were collected and randomly allocated into nine groups, each containing five biological replicates.

### 2.2. Exposure to SPI (Ethyl Polymyxin)

About 60 g/L SPI was diluted to concentration gradients of 0.8, 0.4, 0.2, 0.1, 0.05, 0.025, 0.0125, and 0.00625 mg/L using a 50% sucrose solution. Before the experiment, the experimental bees were subjected to starvation and were treated with SPI for 2 h. A total of 60 g/L of SPI was provided from Dow AgroSciences. A five milliliter mixture of sucrose–SPI with varying concentration gradients was introduced into the plastic feeding box. For consumption, a 50% sucrose solution was added, allowing for ad libitum feeding. The sucrose solution was replaced every 12 h, dead bees were removed, and toxicity curves were generated using the Statistical Package for the Social Sciences (SPSS- https://www.ibm.com/products/spss-statistics, accessed on 1 January 2025) [32] over 48 h. The outcomes of the acute toxicity assessment are summarized in Table 1.

Following the acute toxicity assessment, bees were administered at LC20 and LC50 concentrations of SPI, and the midgut morphology of their adults was examined and documented under a light microscope after 48 h of SPI treatment (Nikon SMZ800N microscope system—Nikon, Tokyo, Japan). Briefly, the adult honey bees from LC20 and LC50 were chilled in a refrigerator before being transversely sectioned at the anterior and posterior regions. The sectioning was performed on a dissecting plate containing 4% paraformaldehyde in 0.1 M of sodium phosphate buffer (pH = 7.4). The samples were fixed overnight at a chilling temperature in the fixative solution. Subsequently, the honey bees were washed with 0.1 M sodium phosphate buffer and then subjected to dehydration in ethanol series (70–100%). In the follow-up experiment, the samples were embedded in resin and histological sections were prepared at thicknesses of 5 μm. Finally, the slides were examined under a light microscope (Nikon SMZ800N microscope system—Nikon, Tokyo, Japan).

### 2.3. Enzyme Activity Assay

Ten adult Chinese honey bees (*Apis cerana cerana* Fabricius) were chosen from each treatment group, and 0.1 g of the midgut was put in a 1.5 mL enzyme-free EP tube for homogenization. Nine volumes of pre-cooled 0.1% phosphate buffer (pH = 7.4) were added to the EP tube to make 1 mL, after which the tube was agitated for 5 min at 60 Hz, followed by centrifugation at 4 °C and 12,000 r/min for 10 min. The supernatant was collected, and the enzymatic activities of cytochrome P450, glutathione S-transferase, carboxylesterase, and acetylcholinesterase were assessed following the ELISA kit instructions (Feiyue bio, Wuhan, China) using a microplate reader (SpectraMax ABS plus, Molecular Devices, Silicon Valley, CA, USA) by Norminkoda Biotechnology Co., Ltd., Wuhan, China). Five biological replicates were carried out for each treatment.

### 2.4. RNA Extraction and Sequencing

The transcriptome analysis was performed on honey bee pupae subjected to the aforementioned treatment. Five biological replicates were acquired for each experimental group. RNA was extracted with a commercially available RNA extraction kit (Jiangsu CoWin Biotech Co., Ltd., Taizhou, China) according to the manufacturer’s instructions. The RNA quality and concentration were assessed utilizing a Bioanalyzer machine to verify the integrity of the samples before sequencing. High-throughput sequencing was conducted utilizing the Illumina NovaSeq/DNBSEQ-T7 platforms (San Diego, CA, USA), generating raw data in the FASTQ format that included sequence information and corresponding quality scores.

### 2.5. Data Quality Control, Filtering, and Analysis

The raw sequencing data were subjected to quality control by FastQC (version 0.11.5) [33]. This phase encompassed the assessment of the base quality at all positions within the reads, nucleotide distribution mapping, and the quantification of the ‘N’ base content (unknown bases). Following the initial quality assessment, raw reads were filtered to exclude sequences with low-quality scores or adaptor contamination using Fastp (version 0.20.0) [34]. The cleaned, high-quality reads were subsequently aligned to the *Apis cerana* reference genome (NCBI Genbank accession number GCA_029169275.1, https://www.ncbi.nlm.nih.gov/datasets/genome/GCF_029169275.1/, accessed on 1 January 2025) using HISAT2 (version 2.0.1-beta) [35], with alignment metrics including total reads, mapped reads, and mapping rates calculated to evaluate alignment effectiveness. Subsequently, gene expression levels were evaluated using the feature Counts (version 1.6.0) [36], and expression values were normalized by the Fragments Per Kilobase of exon per Million reads mapped (FPKM) method [37] for inter-sample comparison. To find significant changes in expression between treatment groups (e.g., control vs. SPI exposure), differentially expressed genes (DEGs) were analyzed using the DESeq2 R Package (https://github.com/thelovelab/DESeq2, accessed on 1 January 2025) [38], with a false discovery rate (FDR) threshold of <0.05. Subsequently, gene ontology (GO) enrichment [39] and Kyoto Encyclopedia of Genes and Genomes (KEGG) pathway analyses [40] were performed to investigate the biological functions and metabolic pathways affected by SPI exposure, with statistical analyses conducted using the R software for all quantitative assessments.

## 3. Results

### 3.1. The Identification of Differentially Expressed Genes in the Pupae of Apis cerana cerana Fabricius in Response to SPI

High-throughput paired-end transcriptome sequencing was conducted on the following groups on the Illumina platform: control group (CK), lethal concentration 20 (LC_20_), and lethal concentration 50 (LC_50_). Sequencing analysis indicated that the three biological replicates of each sample yielded an average of 51.76 million reads for the CK group, 50.78 million reads for the LC_20_ group, and 50.15 million reads for the LC_50_ group (Table 2). Upon the removal of adapters and substandard tags, the CK group yielded an average of 51.73 million clean reads, whereas the LC20 group produced 50.74 million, and the LC_50_ group generated 50.28 million. The clean reads from three groups demonstrated an average GC content of 38.91%, with Q20 and Q30 scores of 99.66% and 98.63%, respectively. These results indicate that the quality of the RNA sequencing profile for each biological replicate was sufficiently high to detect the DEGs. The clean reads were aligned to the reference genome (NCBI GenBank accession number: GCA_029169275.1) and exhibited an average mapping rate of 97.87% across all sample groups.

**Figure 1:**
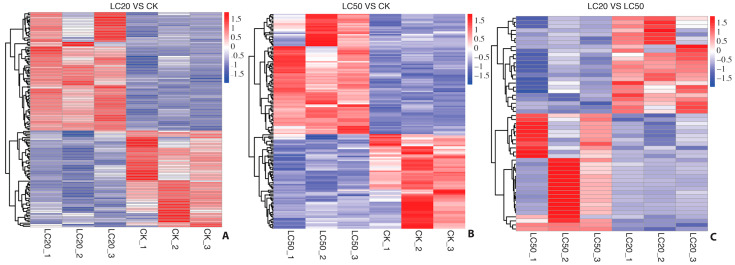
Heatmap display of differentially expressed genes in all 3 comparisons. Hierarchical clustering analysis was conducted on the identified differentially expressed genes. Panel (**A**) represents the LC_20_ vs. control groups, panel (**B**) represents the LC_50_ vs. control groups, while panel (**C**) represents the differential expression for both treatments.

**Figure 2:**
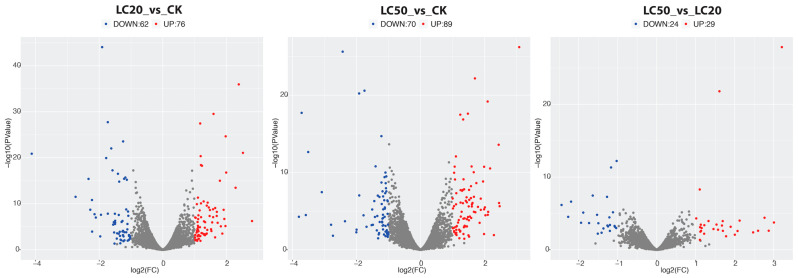
Differential gene volcano plot of all three comparisons. Scatterplots were created for all differentially expressed genes, with upregulated genes depicted in red and downregulated genes in blue.

Based on the criteria of an absolute log_2_FC value exceeding 1 and a raw *p*-value below 0.05, DEGs were identified in the following group comparisons (Table 3): LC_20_ vs. CK (138 DEGs, 79 upregulated and 62 downregulated), LC50 vs. CK (159 DEGs, 89 upregulated and 70 downregulated), and LC_50_ vs. LC_20_ (53 DEGs, 29 upregulated and 24 downregulated). The heat map and scatter plot analysis indicated a significant difference in gene expression patterns among treated and control groups (Figure 1 and Figure 2). A comprehensive list of DEGs is provided in the Supplementary Materials File S1.

### 3.2. Identification of Unique Genes Affected in SPI-Exposed Honey Bee Pupae

**Figure 3:**
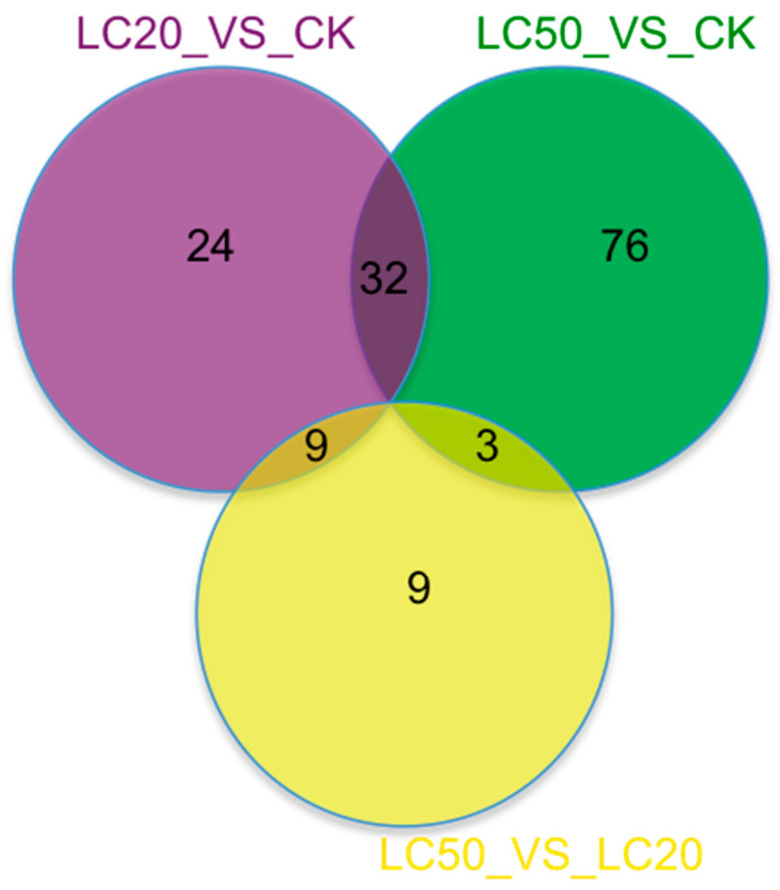
A Venn diagram highlighting the unique and overlapping DEGs across the following comparison groups: LC_20_ vs. CK_LC_50_ vs. CK_LC_50_ vs. LC_20_. The unique and shared DEGs can be seen in the Venn diagram.

A Venn diagram was constructed to examine the overlap and uniqueness of DEGs identified among the experimental groups (Figure 3). A total of 32 differentially expressed genes were identified in both the LC_20_ versus CK and the LC_50_ against CK comparisons. In addition to the overlapping differentially expressed genes, 24 particular DEGs were identified in the comparison of LC_20_ to CK, whereas 76 distinct DEGs were observed in the comparison of LC_50_ to CK. Furthermore, nine DEGs were found to overlap in the comparison of LC_20_ versus CK and LC_50_ versus LC20 in response to SPI, whereas the unique DEGs were 24 for LC20 versus CK and nine for LC_50_ versus LC_20_. The comparison of LC_50_ against CK and LC_50_ against LC_20_ identified three overlapping DEGs, with 76 unique DEGs for LC50 versus CK and nine specific DEGs for LC_50_ versus LC_20_.

### 3.3. GO Enrichment Analysis of Significant DEGs

The number of genes linked to GO annotations was quantified and categorized into three functional classifications: molecular function (MF), cellular component (CC), and biological process (BP). Notable DEGs were further analyzed for GO enrichment to classify each comparison within these functional categories.

#### 3.3.1. LC_20_ vs. CK

**Figure 4:**
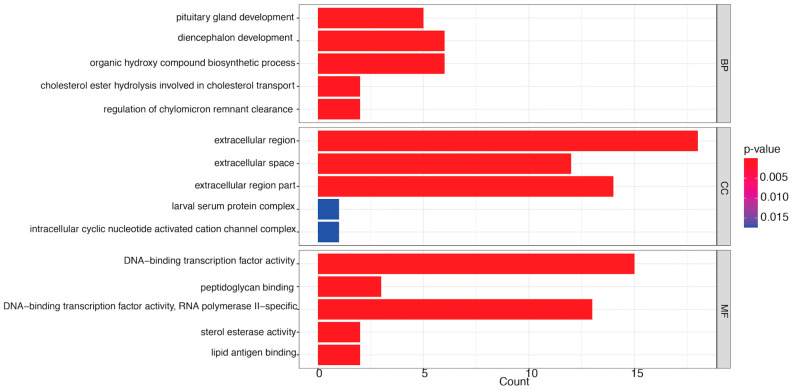
Top gene ontology (GO) terms (*p* < 0.05) enriched in LC20 vs. CK comparison in the categories of molecular function (MF), cellular component (CC), and biological process (BP). The abscissa represents the description of the enriched GO number, while the ordinate indicates the proportion of enrichment. This result is derived by dividing the number of enriched genes by the total number of annotated genes within that specific GO category in the genome. Colors denote importance, with darker hues signifying a greater degree of enrichment.

This comparison influenced several MF terms, including DNA-binding transcription factor activity, peptidoglycan binding, RNA polymerase II-specific, sterol esterase activity, and lipid antigen binding (Figure 4). Transcription factors (TFs) are essential for regulating gene expression, acting as crucial intermediaries that decode genetic regulatory signals. They attach to particular DNA sequences in promoters and enhancers, either promoting or obstructing the recruitment of RNA polymerase II to target genes. This interaction is essential for the accurate regulation of transcription initiation, guaranteeing that genes are expressed at the appropriate moment and within the suitable cellular environment [41]. Moreover, esterases catalyze the hydrolysis of molecules containing ester, amide, and thioester linkages, facilitating prodrug activation or detoxification [42]. Finally, the binding of lipid antigens is crucial for immune recognition and response [43]. Thus, collectively, these functions highlight the complex molecular pathways that underpin honey bee health and adaptability.

Further, CC terms predominantly highlighted the pupal serum protein complex and the intracellular cyclic nucleotide-activated cation channel complex. Cyclic nucleotide-gated (CNG) ion channels are pivotal in vision and olfaction, producing electrical responses to light in photoreceptors and to odorants in olfactory receptors [44].

Furthermore, chemicals such as methylglyoxal augment the antimicrobial properties of honey. These organic compounds enhance honey’s nutritional content and provide health benefits, such as antioxidant and anti-inflammatory properties [45,46]. In addition, cholesterol esters are vital in insect physiology, functioning as storage forms of sterols necessary for cellular membrane integrity and steroid hormone synthesis [47]. Lastly, chylomicron remnants are crucial for sustaining lipid homeostasis and energy equilibrium [48]. The genes linked to each enriched GO term are enumerated in Supplementary File S2.

#### 3.3.2. LC_50_ vs. CK

**Figure 5:**
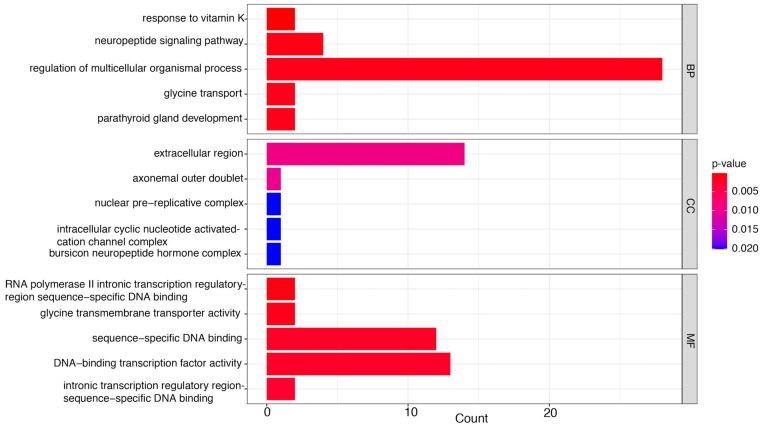
The most substantially enriched gene ontology (GO) terms (*p* < 0.05) in the comparison between LC50 and CK. The findings are presented for the three primary GO categories: molecular function (MF), cellular component (CC), and biological process (BP). The *y*-axis displays the description of each enriched GO term. The *y*-axis, denoting the proportion of enrichment, illustrates the ratio of DEGs associated with a particular GO term to the total number of genes annotated with that GO term within the genome. The *x*-axis denotes the number of enriched genes corresponding to each GO term. The color scale denotes the extent of enrichment, with darker colors signifying a higher level of statistical significance in the enrichment observed.

The MFs identified in this comparison encompassed RNA polymerase II intronic transcription regulatory region sequence-specific DNA binding, glycine transmembrane transporter activity, sequence-specific DNA binding, DNA-binding transcription factor activity, and intronic transcription regulatory region sequence-specific DNA binding (Figure 5).

The glycine transporter in honey bees may play a role in neural functions associated with temperature regulation and stress responses, particularly concerning the involvement of the *Apis cerana* vesicular inhibitory amino acid transporter (AcVIAAT) gene in adapting to extreme temperatures [49]. Secondly, intronic transcription regulatory areas of RNA polymerase II facilitate sequence-specific DNA binding, affecting transcriptional regulation by recruiting transcription factors and altering polymerase activity. These interactions are essential for gene expression and alternative splicing, facilitating appropriate cellular responses to developmental and environmental stimuli [50].

Equally important are the CC terms, which include the axonemal outer doublet, the nuclear pre-replicative complex, the intracellular cyclic nucleotide-activated cation channel complex, and the bursicon neuropeptide hormone complex. The axonemal outer doublets are crucial elements of the ciliary and flagellar structures in honey bees, enabling movement and locomotion [51]. Moreover, bursicon is a neuropeptide hormone in insects that plays a vital part in cuticle tanning, which includes the processes of melanization and sclerotization, in addition to wing expansion after molting [52].

Moving on, the BP terms pertain to the response to vitamin K, neuropeptide signaling pathways, glycine transport, and the regulation of multicellular organismal processes, which are related to food-related behavior and stress responses. In these impacted BP terms, vitamin K is linked with improvement of brood rearing, hence promoting colony growth and health [53], which is essential for numerous physiological functions, including muscular activity, brain communication, and general well-being [54,55].

#### 3.3.3. LC_50_ vs. LC_20_

**Figure 6:**
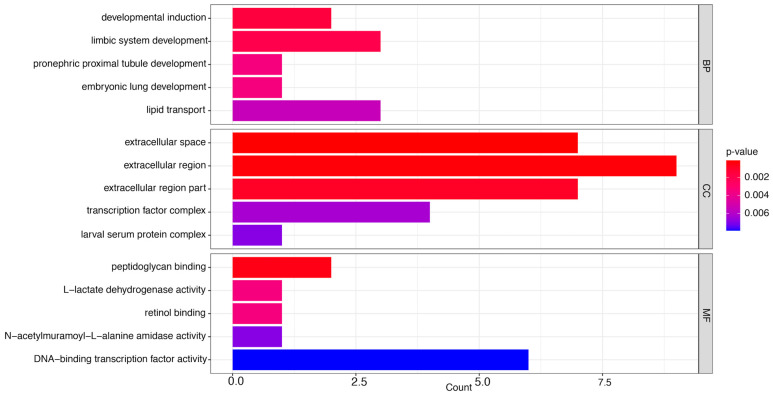
Gene ontology (GO) enrichment analysis of differentially expressed genes in LC50 compared to LC20. The most significantly enriched GO terms are presented, classified into the categories of molecular function (MF), cellular component (CC), and biological process (BP). The bars illustrate the ratio of enriched genes associated with each GO term, calculated as the number of enriched genes divided by the total number of annotated genes corresponding to that GO term. The *x*-axis denotes the number of enriched genes corresponding to each GO term. Higher enrichment scores are shown by darker colors.

This comparison included MF terms related to DNA-binding transcription factor activity, N-acetylmuramoyl-L-alanine amidase activity, retinol binding, L-lactate dehydrogenase activity, and peptidoglycan binding (Figure 6). L-lactate dehydrogenase functions as a critical regulator of gluconeogenesis and DNA metabolism [56].

Peptidoglycan binding in honey bees, facilitated by Peptidoglycan Recognition Proteins (PGRPs), is essential for innate immunity since it identifies bacterial pathogens and activates the Toll signaling cascade. This mechanism stimulates antimicrobial peptide synthesis, which is vital for protecting against infections and sustaining colony health [57,58].

Furthermore, the CC terms encompassed transcription factor complex and pupal serum protein complex. Pupal serum proteins in honey bees, especially those present in the hemolymph, are essential for development and nutrition. During the pupal development, notable alterations in protein expression occur, characterized by a substantial rise in protein transporters and food reserves that facilitate development and immunological function. Specific pupal serum proteins participate in immunological responses, with prophenoloxidase exhibiting a positive correlation with stages of pupal development [59].

Finally, the BP impacted developmental induction, limbic system development, pronephric proximal tubule development, embryonic lung development, and lipid transport. Embryonic lung development in honey bees involves the establishment of a tracheal respiratory system, occurring approximately 45 h post-fertilization, resulting in the creation of air-filled tubes for gas exchange. This system facilitates respiration and metabolic needs during their entire lifecycle [60].

These disturbances can undermine cellular integrity, signaling pathways, and overall physiological health, significantly impairing bees’ capability to execute vital functions and jeopardizing their survival.

### 3.4. KEGG Pathway Enrichment Analysis of Significant DEGs

#### 3.4.1. LC_20_ vs. CK

**Figure 7:**
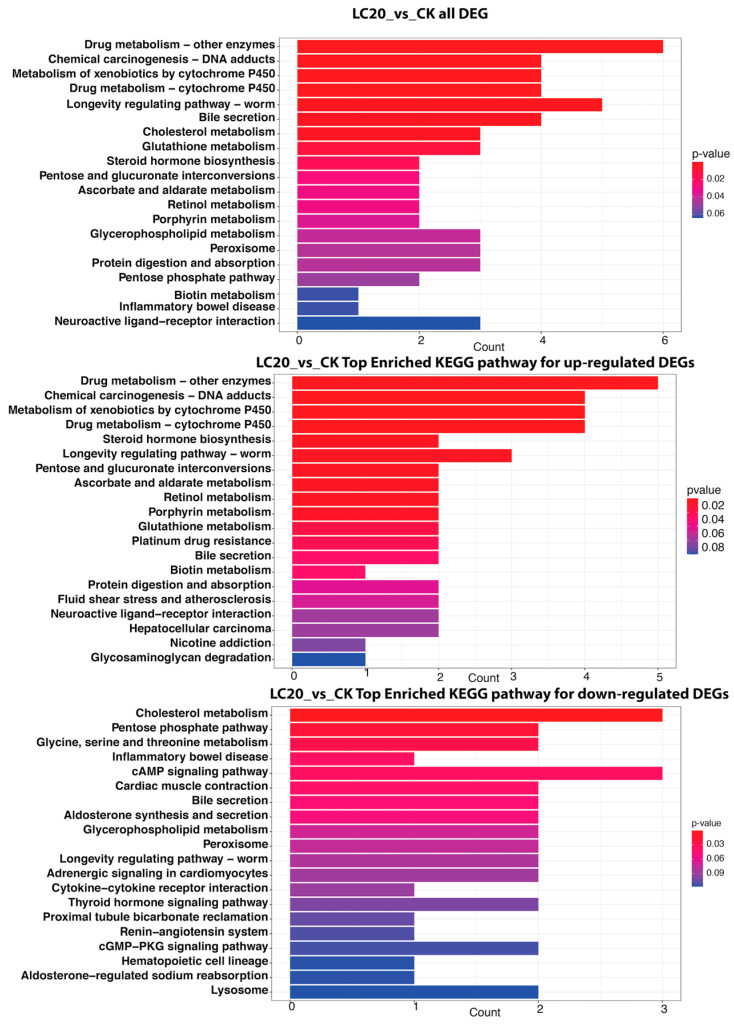
The upper panel represents the top enriched KEGG pathways for all DEGs in the comparison of LC20 and CK. The *x*-axis denotes the count of genes linked to each pathway, while the *y*-axis illustrates the KEGG pathway term. The middle panel represents the top enriched KEGG pathways for up-regulated DEGs in the LC20 vs. CK comparison. The bottom panel represents the top enriched KEGG pathways for down-regulated DEGs in the LC20 vs. CK comparison.

The KEGG pathway enrichment investigation comparing the LC20 group to the CK revealed significant alterations in metabolic pathways in honey bees exposed to SPI (Figure 7). Among the DEGs, pathways such as “Drug metabolism—other enzymes”, “Chemical carcinogenesis—DNA adducts”, and “Metabolism of xenobiotics by cytochrome P450” were significantly enriched, reflecting an enhanced response to detoxification mechanisms. The “Drug metabolism—other enzymes” route comprised six DEGs, but the “Chemical carcinogenesis—DNA adducts” and “Metabolism of xenobiotics by cytochrome P450” pathways each contained four DEGs, highlighting the potential genotoxic risks linked to SPI exposure. Furthermore, pathways associated with longevity regulation and bile secretion were enriched, indicating wider physiological implications. On the contrary, down-regulated pathways encompassed “Cholesterol metabolism” and the “Pentose phosphate pathway”, signifying disturbances in vital metabolic processes that may jeopardize honey bee health.

#### 3.4.2. LC_50_ vs. CK

**Figure 8:**
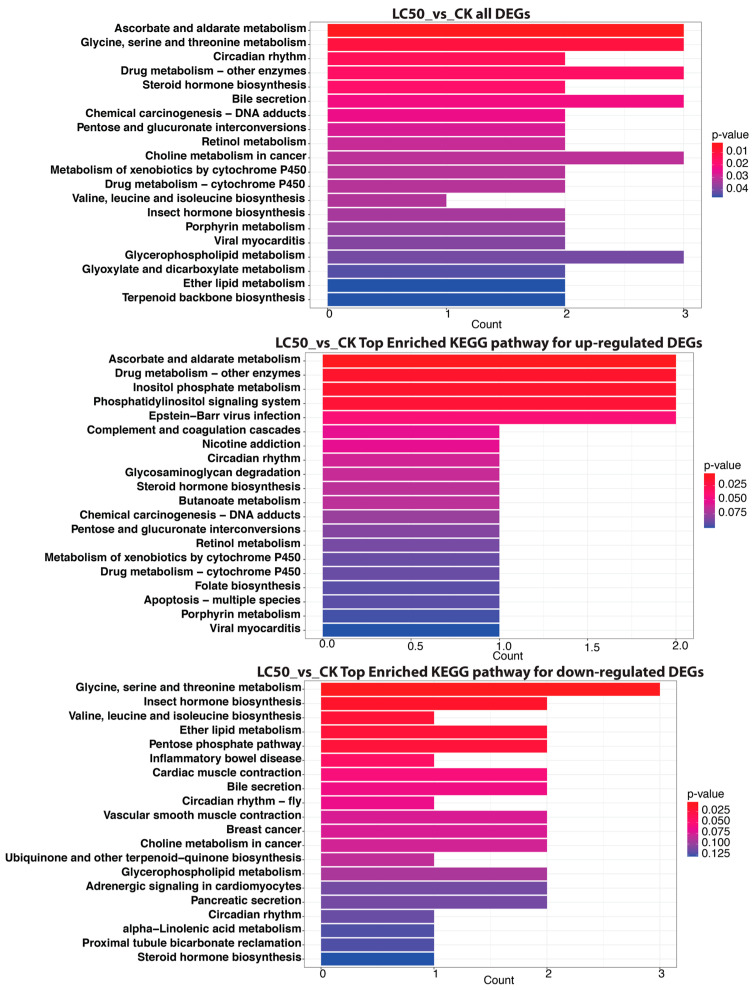
KEGG pathway analysis demonstrates unique enrichment patterns for all DEGs in LC50 compared to CK (Top panel). The middle panel represents the KEGG pathway analysis, which highlights distinct enrichment profiles for upregulated DEGs in LC_50_ compared to CK. The bottom panel represents KEGG pathway analysis enrichment for down-regulated DEGs in LC_50_ compared to CK. The count of genes corresponding to each KEGG pathway (*x*-axis) is shown for each enriched pathway (*y*-axis).

The KEGG pathway enrichment analysis contrasting the LC_50_ group with the control group (CK) indicated significant modifications in metabolic and physiological pathways in honey bees subjected to SPI exposure (Figure 8). Significantly, pathways including “Ascorbate and aldarate metabolism”, “Glycine, serine and threonine metabolism”, and “Drug metabolism—other enzymes” were enriched among all differentially expressed genes, each comprising three DEGs, suggesting disturbances in metabolic detoxification and amino acid metabolism. The investigation additionally found up-regulated pathways, such as “Ascorbate and aldarate metabolism” and “Drug metabolism—other enzymes”, indicating an enhanced potential for detoxification and oxidative stress response. In contrast, down-regulated pathways like “Glycine, serine and threonine metabolism” and “Insect hormone biosynthesis” suggest a suppression of essential metabolic activities and hormonal regulation, potentially affecting honey bee health.

#### 3.4.3. LC_50_ vs. LC_20_

**Figure 9:**
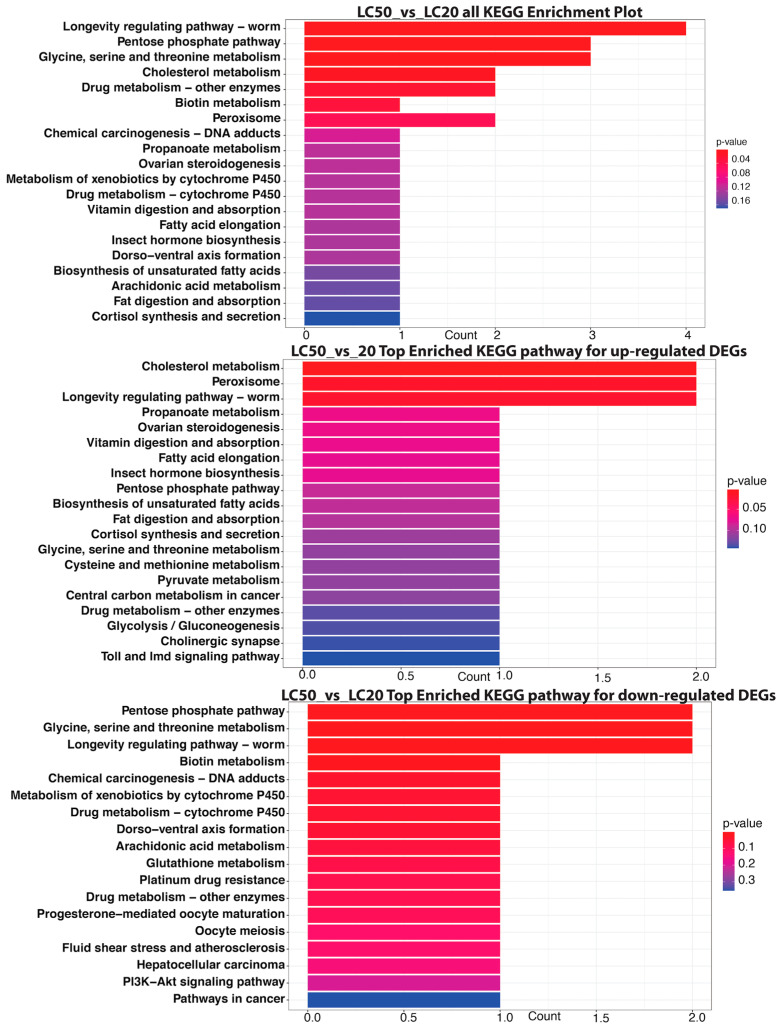
Significant KEGG pathways influenced by differential gene expression in LC_50_ relative to LC_20_ for all DEGs (top panel): *x*-axis: gene count; *y*-axis: KEGG pathway. Top KEGG pathways substantially enriched in up-regulated genes between LC_50_ and LC_20_ (Middle panel). Significantly enriched KEGG pathways among down-regulated DEGs in the LC_50_ vs. LC_20_ comparison (bottom panel). Each pathway’s gene count is shown in a bar plot, where the color denotes the statistical significance (*p*-value). KEGG enriched statistics for all three comparisons (LC_20_ vs. CK, LC_50_ vs. CK, and LC_50_ vs. LC_20_) are provided in the Supplementary File S3.

When comparing the LC_50_ group to the LC_20_ group using KEGG pathway enrichment analysis, the honey bees exposed to SPI showed notable changes in their metabolic pathways (Figure 9). Among all differentially expressed genes, “Longevity regulating pathway—worm”, “Pentose phosphate pathway”, and “Glycine, serine and threonine metabolism” were significantly enriched, signifying disturbances in metabolic control and longevity processes. The “Longevity regulating pathway” was notably significant, encompassing four DEGs, whereas the “Pentose phosphate pathway” and “Glycine, serine and threonine metabolism” each had three DEGs, indicating a possible influence on energy metabolism and amino acid synthesis. Upregulated pathways, including “Cholesterol metabolism” and “Peroxisome”, signify enhanced lipid processing and cellular metabolism, whereas downregulated pathways such as the “Pentose phosphate pathway” and “Chemical carcinogenesis—DNA adducts” suggest impaired metabolic functions and possible genotoxic risks linked to SPI exposure.

### 3.5. Morphological Changes in Midgut of Apis cerana cerana Fabricius

**Figure 10:**
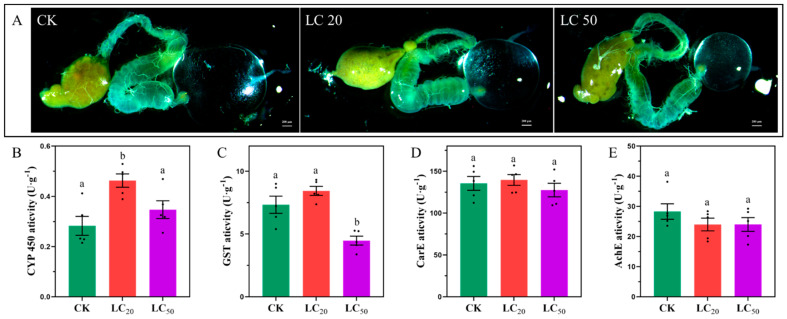
The impact of SPI on the midgut and midgut detoxifying enzymes of *Apis cerana cerana* Fabricius. (**A**). Representative pictures of the midgut of healthy and SPI-infested *Apis cerana cerana* Fabricius (bars, 200 μm). (**B**). The impact of varying SPI concentrations on the CYP 450 activity of *Apis cerana cerana* Fabricius (one-way ANOVA, Tukey’s HSD test, F2,12 = 7.407, *p* = 0.008). (**C**). Effects of different SPI concentrations on the GST activity of *Apis cerana cerana* Fabricius (one-way ANOVA, Tukey’s HSD test, F2,12 = 17.374, *p* = 0.0003). (**D**). Effects of different SPI doses on the AChE activity of *Apis cerana cerana* Fabricius (one-way ANOVA, Tukey’s HSD test, F2,12 = 1.138, *p* = 0.353). The differences between the means (±SE) in (**B**–**E**) were evaluated using one-way ANOVA, followed by Tukey’s HSD test (values with different letters a,b show significant differences).

In the CK group, the midgut exhibited a normal morphology with an intact structure and fluorescence. The midgut structure shows noticeable changes in the LC_20_ group, including possible enlargement or swelling, partial distortion, and loss of integrity. This disarray signifies moderate impairment. The LC_50_ group demonstrated more pronounced damage, characterized by significant midgut degeneration, tissue disintegration, and structural disruption. Figure 10 comprehensively illustrates the detrimental effects of SPI exposure on both the midgut structure and the detoxification enzyme systems. It reveals striking dose-dependent morphological damage to the midgut, where the control group displays intact tissue architecture with clearly defined luminal borders and uniform epithelial cells. In contrast, the LC_20_ group exhibits moderate yet significant structural alterations, including epithelial swelling and partial lumen distortion, indicating initial stress responses. The LC_50_ group shows severe degeneration characterized by complete tissue disintegration and ruptured cellular integrity, demonstrating SPI’s capacity to cause irreversible organ damage. These morphological changes directly correlate with the enzymatic responses shown in Panels B-E. The cytochrome P450 (P450) and glutathione S-transferase (GST) activities (Panels B and C) initially increase at LC_20_ (*p* < 0.008 and *p* < 0.0003, respectively), reflecting the pupae’s attempt to detoxify SPI, but sharply decline at LC_50_ as the system becomes overwhelmed. Carboxylesterase (CarE) activity (Panel D) shows minimal response, suggesting it plays a secondary role in SPI metabolism. Most notably, acetylcholinesterase (AChE) activity (Panel E) is significantly inhibited at both concentrations (*p* < 0.05), with more pronounced effects at LC_50_, confirming SPI’s neurotoxic action through cholinergic disruption. Together, these findings demonstrate a clear progression from adaptive responses at sublethal doses to systemic failure at lethal concentrations, with midgut damage and enzyme inhibition mutually exacerbating SPI’s toxic effects. The morphological and biochemical data are further supported by transcriptomic results showing corresponding changes in detoxification and stress-response pathways, providing a comprehensive view of SPI’s multifaceted toxicity in honey bee pupae.

### 3.6. Enzyme Activity Assay

Enzymes that safeguard honey bees in pesticide-laden environments are regarded as sensitive biomarkers, particularly those associated with detoxification processes and oxidative stress. Consequently, the enzymatic activities previously regarded as indicators for xenobiotic exposure in honey bees were also evaluated [3,8,61]. As illustrated in Figure 10B–E, the enzyme detoxification experiment on honey bees subjected to SPI demonstrated notable changes in enzymatic activity. Cytochrome P450 (P450) activity considerably increased at LC_20_ (*p* < 0.05), indicating an elevation of this enzyme as a component of the midgut detoxification process. Nonetheless, P450 activity diminished around LC_50_, indicating possible enzyme inhibition resulting from severe toxic stress. The activity of GST exhibited a notable rise at LC 20 (*p* < 0.05), indicating an adaptive response to oxidative stress; however, it dramatically decreased by LC_50_, suggesting enzyme inhibition or a reduction in detoxification capacity. CarE activity exhibited a comparable pattern, showing a significant increase at LC20 (*p* < 0.05), but no further rise at LC_50_, possibly attributable to enzyme saturation or midgut impairment. The activity of AChE showed a significant decrease at both LC_20_ and LC_50_ (*p* < 0.05), with a more substantial reduction observed at LC_50_, suggesting possible neurotoxicity from SPI use.

## 4. Discussion

The findings of this study provide evidence that SPI exposure induces significant physiological and molecular disruptions in *Apis cerana cerana* pupae, with dose-dependent effects on midgut morphology, detoxification enzyme activity, and gene expression patterns. Our results offer novel insights into the mechanisms of SPI toxicity in Asian honey bees and have important implications for pollinator conservation in agricultural landscapes.

Our comprehensive analysis reveals significant dose-dependent effects of Spinetoram (SPI) on honey bee physiology through integrated transcriptomic and biochemical investigations. The GO enrichment analysis demonstrated distinct molecular responses across exposure levels, with LC_20_ concentrations inducing adaptive mechanisms through the upregulation of DNA-binding transcription factors and lipid antigen-binding proteins (Figure 4). These changes suggest coordinated activation of cellular stress responses and immune recognition pathways to counteract pesticide exposure [43,62]. At lethal LC_50_ concentrations, we observed markedly different molecular signatures characterized by the dysregulation of glycine transporters and RNA polymerase II binding activity (Figure 5). These alterations indicate substantial neural and transcriptional disruption, particularly through interference with neuropeptide signaling pathways that regulate critical colony behaviors [63,64,65]. The observed changes in glycine transport may reflect neurotransmitter imbalance, consistent with SPI’s known action on nicotinic acetylcholine receptors [13,19].

KEGG pathway analysis provided compelling evidence of metabolic trade-offs during SPI detoxification. While both concentrations activated xenobiotic metabolism pathways (“Drug metabolism—other enzymes”, “Ascorbate and aldarate metabolism”), LC50 exposure uniquely suppressed essential metabolic processes, including amino acid metabolism (“Glycine, serine and threonine metabolism”), hormone biosynthesis (“Insect hormone biosynthesis”), and energy production (“Pentose phosphate pathway”). This pattern suggests a threshold beyond which detoxification demands overwhelm metabolic capacity [8,66]. The simultaneous upregulation of longevity-associated pathways at LC_50_ may represent a failed compensatory mechanism to mitigate cellular damage [67].

Our enzymatic analyses revealed a characteristic biphasic response in detoxification systems. The initial activation of cytochrome P450 (*p* = 0.008) and glutathione S-transferase (*p* = 0.0003) at LC_20_ reflected robust phase I and II detoxification efforts [68,69]. However, the subsequent inhibition at LC50 concentrations indicates system overload, potentially through cofactor depletion (NADPH, glutathione), direct enzyme inhibition, and irreversible cellular damage [3,15]. The persistent inhibition of acetylcholinesterase across all exposure levels (*p* < 0.05) confirms SPI’s neurotoxic potential, with particular concern for developmental and behavioral consequences in adult bees [3,19]. These findings align with previous reports of spinosyn-induced neurotoxicity in insect models [20,25].

These findings carry important implications for pollinator conservation and pesticide regulation. The clear dose-dependent progression from compensatory responses to systemic failure, particularly across multiple organ systems, suggests that current risk assessment protocols may be underestimating SPI’s sublethal effects on honey bee pupae. The threshold between LC_20_ and LC_50_ concentrations appears particularly critical, representing a transition point where detoxification systems become overwhelmed. Furthermore, the simultaneous impacts on digestive, metabolic, and neural functions could have compounding effects at the colony level that warrant further investigation.

Several important questions remain for future research. The long-term colony consequences of pupal SPI exposure merit particular attention, as does the potential for synergistic effects with other environmental stressors. Investigation of genetic variation in detoxification capacity among different honey bee populations could help explain the observed differences in pesticide sensitivity. Additionally, the development of more sensitive biomarkers for SPI exposure monitoring would greatly enhance field-level risk assessment capabilities.

The severe midgut damage observed at LC_50_ concentrations (Figure 10A) represents more than just tissue degeneration—it reflects a fundamental breakdown in the pupa’s primary interface for nutrient absorption and xenobiotic processing. The progression from epithelial swelling at LC_20_ to complete tissue disintegration at LC_50_ suggests that SPI may induce programmed cell death pathways, as demonstrated in other insects exposed to spinosyns [20,70]. This structural damage likely compromises multiple physiological functions simultaneously: (1) impaired digestion and nutrient uptake, (2) reduced metabolic capacity, and (3) compromised barrier function against pathogens and toxins. The midgut’s role as both a digestive organ and immune tissue [57,58] means these morphological changes could have cascading effects on pupal development and disease resistance.

The biphasic response of detoxification enzymes reveals a critical threshold in the bees’ capacity to metabolize SPI. The initial upregulation of P450 and GST activities at LC_20_ represents a robust but ultimately insufficient defense mechanism. This aligns with transcriptome data showing the enrichment of drug metabolism pathways at sublethal doses (Figure 7 and Figure 10). However, the subsequent enzyme inhibition at LC_50_ suggests either direct inactivation of enzymes by SPI or depletion of essential cofactors (e.g., glutathione).

## Figures and Tables

**Table 1:** Toxicity of SPI to *Apis cerana cerana* Fabricius.

Insecticide	Toxicity Regression Equation	LC_20_ (mg·L^−1^)	95% Confidence Interval	LC_50_ (mg·L^−1^)	95% Confidence Interval	R^2^
SPI	y = 1.043 x + 0.391	0.066	0.044 ~ 0.092	0.421	0.290 ~ 0.701	0.966

**Table 2:** A summary of transcriptome data indicating the quantity of reads, sequence quality, and the mapping rate.

Sample	Raw Reads	Raw Bases	Clean Reads	Clean Bases	Clean Ratio	Q20	Q30	GC	Mapped Reads	Map Rate	Uniq
CK_1	49,289,180	7,393,377,000	49,279,482	7,339,884,868	99.98%	99.60%	98.40%	39.37%	48,264,825	97.94%	47,429,902
CK_2	54,338,544	8,150,781,600	54,324,974	8,055,938,328	99.98%	99.63%	98.54%	40.48%	53,104,962	97.75%	52,246,861
CK_3	51,610,170	7,741,525,500	51,598,260	7,708,607,852	99.98%	99.41%	97.72%	39.41%	50,432,130	97.74%	49,526,790
LC20_1	55,398,272	8,309,740,800	55,384,436	8,280,072,910	99.98%	99.40%	97.72%	39.39%	54,172,283	97.81%	53,181,246
LC20_2	57,170,342	8,575,551,300	57,158,026	8,517,249,552	99.98%	99.63%	98.52%	38.91%	55,886,945	97.78%	54,938,014
LC20_3	40,079,318	6,011,897,700	40,070,638	5,947,681,080	99.98%	99.62%	98.49%	39.19%	39,209,575	97.85%	38,644,603
LC50_1	61,038,878	9,155,831,700	61,026,728	9,092,782,540	99.98%	99.60%	98.40%	39.27%	59,609,971	97.68%	58,498,542
LC50_2	48,208,610	7,231,291,500	48,197,838	7,179,127,732	99.98%	99.63%	98.52%	38.94%	47,156,526	97.84%	46,412,287
LC50_3	41,712,970	6,256,945,500	41,703,366	6,235,278,786	99.98%	99.45%	97.86%	39.27%	40,661,149	97.50%	39,975,945

**Table 3:** Number of DEGs identified in three comparison groups.

SampleID	Total	UP	DOWN
LC_20__vs_CK	138	76	62
LC_50__vs_CK	159	89	70
LC_50__vs_LC_20_	53	29	24

## Data Availability

The data sets supporting the findings of this work are included within the article and its Supplementary Materials (Files S1–S3). Additional information may be obtained from the corresponding author.

## Supplementary Materials

The following supporting information can be downloaded at: https://www.mdpi.com/article/10.3390/insects16050492/s1, File S1: S1_ Complete List of DEGs (All DEGs, Up-regulated DEGs and Down-regulated DEGs) in all 3 comparisons (LC_20_ vs. CK, LC_50_ vs. CK and LC_50_ vs. LC_20_). This file contains 10 sheets, including 8 data sheets and 1 index sheet; File S2: S2_ List of genes linked to enriched GO terms in all 3 comparisons (LC_20_ vs. CK, LC_50_ vs. CK and LC_50_ vs. LC_20_). There are 19 sheets in this file: 18 data sheets and 1 index sheet; File S3: S3_ KEGG enrichment statistics file. The file consists of 19 sheets, which include 18 data sheets and one index sheet.
