# Supplementary material for: Effect of Spinetoram Stress on Midgut Detoxification Enzyme and Gene Expression of Apis cerana cerana Fabricius
Source: Insects. 2025 May 4;16(5):492. doi: 10.3390/insects16050492 (PMC12111890; doi:10.3390/insects16050492)
Supplement: Supplementary file 1 [file insects-16-00492-s001.zip › Supplementary Data.pdf]

The effect of Spinetoram stress on the midgut detoxification enzyme and genes expression of *Apis cerena* cerana Fabricius

Supplementary file S1

S1_ Complete List of DEGs (All DEGs, Upregulated DEGs and Downregulated DEGs) in all 3 comparisons

(LC20 vs CK, LC50 vs CK and LC50 vs LC20). This file contains 10 sheets, including 8 data sheets and 1 index sheet.

Supplementary file S2

S2_ List of genes linked to enriched GO terms in all 3 comparisons (LC20 vs CK, LC50 vs CK and LC50 vs LC20). There are 19 sheets in this file: 18 data sheets and 1 index sheet.

Supplementary file S3

S3_ KEGG enrichment statistics file. The file consists of 19 sheets, which include 18 data sheets and one index sheet.
